# A Simple Phosphate-Buffered-Saline-Based Extraction Method Improves Specificity of HIV Viral Load Monitoring Using Dried Blood Spots

**DOI:** 10.1128/JCM.00176-17

**Published:** 2017-06-23

**Authors:** A. Tariro Makadzange, F. Kathryn Boyd, Benjamin Chimukangara, Collen Masimirembwa, David Katzenstein, Chiratidzo E. Ndhlovu

**Affiliations:** a Ragon Institute of MGH, MIT and Harvard, Cambridge, Massachussetts, USA; b Department of Medicine, University of Zimbabwe College of Health Sciences, Harare, Zimbabwe; c Department of Virology, National Health Laboratory Service, University of KwaZulu-Natal, Durban, South Africa; d African Institute for Biomedical Sciences and Technology, Harare, Zimbabwe; e Division of Infectious Diseases, Stanford University, Palo Alto, California, USA; Rhode Island Hospital

**Keywords:** HIV, viral load quantification, dried blood spots (DBS), sub-Saharan Africa, cell-free virus elution, viral load monitoring, DBS, dried blood spots

## Abstract

Although Roche COBAS Ampliprep/COBAS TaqMan (CAP/CTM) systems are widely used in sub-Saharan Africa for early infant diagnosis of HIV from dried blood spots (DBS), viral load monitoring with this system is not practical due to nonspecific extraction of both cell-free and cell-associated viral nucleic acids. A simplified DBS extraction technique for cell-free virus elution using phosphate-buffered saline (PBS) may provide an alternative analyte for lower-cost quantitative HIV virus load (VL) testing to monitor antiretroviral therapy (ART). We evaluated the CAP/CTM v2.0 assay in 272 paired plasma and DBS specimens using the cell-free virus elution method and determined the level of agreement, sensitivity, and specificity at thresholds of target not detected (TND), target below the limit of quantification (BLQ) (<20 copies/ml in plasma or <400 copies/ml in DBS), and VL of <1,000 copies/ml, and VL of <5,000 copies/ml. Reported plasma VL ranged from TND, or <20, to 5,781,592 copies/ml, and DBS VL ranged from TND, or <400, to 467,600 copies/ml. At <1000 copies/ml, agreement between DBS and plasma was 96.7% (kappa coefficient, 0.93; *P* < 0.0001). The mean difference between DBS and plasma VL values was −1.06 log_10_ copies/ml (95% confidence interval [CI], −1.17, −0.97; *P* < 0.0001). At a treatment failure threshold of >1,000 copies/ml, the sensitivities, specificities, positive predictive values (PPV), and negative predictive values (NPV) were 92.7%, 100%, 100%, and 94.3%, respectively. PBS elution of DBS offers a sensitive and specific method for monitoring plasma viremia among adults and children on ART at the WHO-recommended threshold of >1,000 copies/ml on the Roche CAP/CTM system.

## INTRODUCTION

More than 15.8 million people infected with HIV are receiving antiretroviral therapy (ART), and the number of people accessing ART has increased by 84% since 2010 (1). A significant proportion of that growth has been through national treatment programs in sub-Saharan Africa. This large-scale public health approach to HIV treatment and care will need to provide lifelong therapy with appropriate clinical and laboratory monitoring to ensure programmatic success (2). In 2013, the WHO introduced routine viral load (VL) monitoring into guidelines for the management of individuals living with HIV in resource- and capacity-limited settings (3). By 2015, only a few countries in sub-Saharan Africa were providing universal access to routine viral load monitoring (4). In Zimbabwe, in 2015, only 5% of the patients in the national ART program had received viral load testing (Ministry of Health and Child Welfare, Zimbabwe [MOHCWZ]).

HIV-1 viral load monitoring of patients on ART is more effective than immunologic monitoring by CD4^+^ T cell count, but has higher incremental cost-effectiveness ratios (5, 6). The cost extends beyond the cost of reagents to include the cost of skilled human resources, sample collection, transportation, processing, infrastructure support, and equipment maintenance. Several diagnostic platforms and assays are available and have been approved for viral quantification (see the WHO list of prequalified *in vitro* diagnostic products, http://www.who.int/diagnostics_laboratory/evaluations/PQ_list/en/). Most of these platforms require centralized laboratories with the appropriate infrastructure and skilled personnel (7), and largely process plasma specimens. The limited capacity to process plasma in remote sites and poor infrastructure for transporting plasma specimens to centralized laboratories has limited the scale-up of routine viral load monitoring with plasma specimens. Dried blood spots (DBS) provide an important alternative to plasma. DBS specimens can be stored for long periods and transported with minimal impact on stability and sample quality (8–10).

The use of DBS for routine viral load monitoring will simplify specimen collection and processing while also leveraging existing equipment and networks for sample transportation that have been established for early infant diagnosis (EID) services. DBS specimens have been successfully utilized in sub-Saharan Africa for molecular-based EID services, with diagnostic sensitivity of 100% and specificity of 99 to 100% (11–13). Although DBS have worked well for DNA quantification in EID, there have been inconsistent results in studies comparing DBS with plasma for quantification of HIV-1 viral RNA for use in routine clinical monitoring of patients on therapy (14).

Viral load values obtained from DBS samples are consistently lower than for plasma (15–17). At low viral load thresholds that define virologic failure, data on performance have conflicted. Low specificity, with an unacceptably high false-positive virologic treatment failure rate from DBS specimens, has been described in several studies (18–21), while other studies using the NucliSens and Abbott assays have shown both high specificity and sensitivity values and are in use for DBS monitoring in some settings (16, 17, 22, 23). The differences across studies may be explained by differences in sample extraction techniques used by different testing platforms, resulting in coextraction and coamplification of viral DNA (9). The COBAS Ampliprep/COBAS TaqMan v2.0 assay (CAP/CTM) (Roche Diagnostics Ltd., Rotkreuz, Switzerland) is a fully automated assay in widespread use in sub-Saharan Africa for EID and virologic monitoring in plasma specimens. The assay is a nucleic acid amplification test for the quantification of HIV-1 RNA in plasma over a range of 20 to 10,000,000 copies/ml. The specimen preextraction (SPEX) reagent includes a chaotrope, guanidium hydrochloride, to inactivate RNases and ensure stability of both viral RNA and total cell-associated viral nucleic acids (12). The low specificity of the DBS assay is due to extraction elution and purification of total nucleic acid. Viral RNA and cell-associated mRNA, integrated provirus, and unintegrated circular episomal DNA account for higher copy numbers in PCR assays from DBS or whole blood in samples with plasma RNA viral loads lower than 1,000 copies/ml (21, 22).

A novel, simple method for the elution of cell-free HIV virus particles from DBS specimens using phosphate-buffered saline (PBS) has been recently developed (24). We used this method for viral RNA extraction, lysis, and reverse transcription from the free virus eluate (FVE) to quantify HIV viral load from dried blood spot, and compared this to plasma samples on the CAP/CTM system. Thresholds for virologic failure, as defined by the WHO, were used to determine the sensitivity and specificity of the assay in DBS specimens (3).

## RESULTS

Paired plasma and DBS sample analysis was done on 272 subjects. The reported results for plasma viral load (VLp) included target not detected (TNDp), BLQp (<20 copies/ml), and quantified values that ranged from 20 to 5,781,592 copies/ml. The reported results for DBS viral load (VLd) included: TNDd, BLQd (<400 copies/ml), and quantified values that ranged from 400 to 467,600 copies/ml. Specimens were defined as TND/BLQ if the result was either TND or BLQ. Among the plasma specimens, 124 (45.6%) were TND/BLQ and 148 (54.4%) were detectable. Among the same specimens, by DBS testing, 158 (58.1%) were TND/BLQ and 114 (41.9%) were quantifiable. Agreement between TND/BLQ and the detectable classification of viral load results for plasma and DBS was seen in 87.5% of the specimens, and the kappa coefficient was 0.75 (standard error [SE] 0.06, *P* < 0.0001). A total of 34 specimens were incorrectly classified as TND/BLQ by DBS; 20 (58.5%) of these were defined as TNDd and 14 (41.2%) as BLQd.

The WHO guidelines define treatment failure as a VL of >1000 copies/ml in plasma and >3,000 to 5,000 copies/ml in DBS (3). In plasma, 123 specimens (45.2%) has a VL greater than 1,000 copies/ml, and 117 (43.0%) had a VL greater than 5,000 copies/ml. By DBS, 114 specimens (41.9%) had a VL greater than 1,000 copies/ml and 83 (30.5%) had a VL greater than 5,000 copies/ml. Agreement between plasma and DBS for defining specimens with VL lower or greater than 1,000 copies/ml was seen in 96.7% of specimens and the kappa coefficient was 0.93 (SE, 0.06; *P* < 0.0001). Agreement between plasma and DBS for defining specimens with VL higher or lower than 5,000 copies/ml was 87.5% and the kappa coefficient was 0.74 (SE, 0.06; *P* < 0.0001).

The risk of virologic failure (>1,000 copies/ml) may be higher among patients with plasma viral load values that are below the limit of quantification (BLQ) than among those in whom the target is not detected (TND) (25, 26). Quantifiable viremia below the WHO threshold of 1,000 copies/ml has been associated with increased risk of virologic failure (25, 27–29). The U.S. treatment guidelines define virologic failure by a lower threshold of detectable viral load greater than 200 copies/ml (29). Although the quantitative threshold of plasma is >20 copies/ml, DBS samples are unable to quantify viremia of less than 400 copies/ml. Misclassification of samples as TND and BLQ by DBS occurred primarily from samples with very low viral copy numbers. Of 34 specimens that were TND or BLQ by DBS, the median plasma VL for these specimens was 66 copies/ml (interquartile range [IQR], 38 to 146); 25 (73.5%) had plasma VL values between 20 to 1,000 copies/ml, and 4 specimens had plasma VL values greater than 200 copies/ml (range, 317 to 939). However, 9 specimens (26.5%) had a plasma VL of >1,000 copies/ml and these were reported as TNDd (*n* = 2) and BLQd (*n* = 7). The two samples that were TNDd by DBS had plasma VL of 1,707 and 34,160 copies/ml. The 7 specimens that were BLQd by DBS had a median VL value of 6,792 copies/ml (IQR, 1,336 to 21,551).

**FIG 1:**
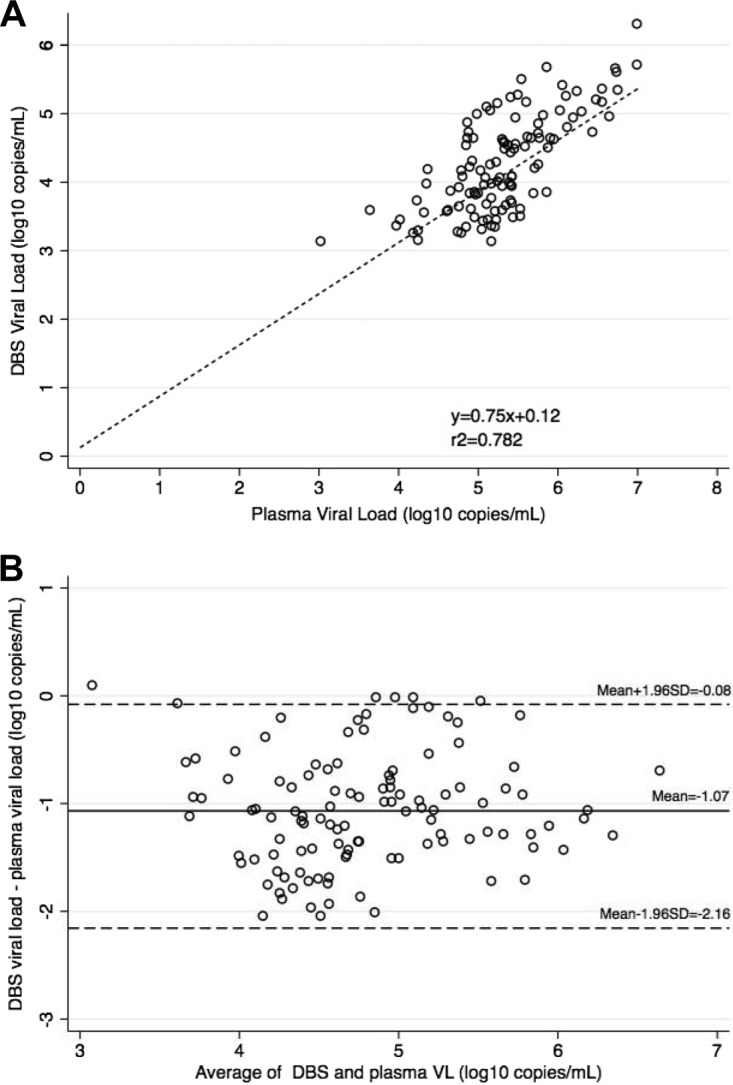
HIV viral load in DBS and plasma samples. (A) Correlation between paired VL measurements obtained from plasma and DBS samples with VL >1000 copies/ml (*n* = 114). Pearson coefficient of determination: r^2^ = 0.782, *P* < 0.0001. (B) Bland-Altman plot showing measurement agreement between DBS and plasma. The mean difference was −1.06 (95% CI, −1.17 to −0.97) log_10_ copies/ml. The limits of agreement (dotted lines) were −2.16 (95% CI, −2.32 to −1.99) and −0.08 (95% CI, −0.25 to 0.09) log_10_ copies/ml.

We evaluated the correlation between viral load values obtained by DBS and those obtained by plasma (Fig. 1a). The correlation coefficient *r*^2^ was 0.782 (*P* < 0.0001). The mean viral load was 4.26 log_10_ copies/ml in DBS and 5.33 log_10_ copies/ml in plasma specimens, with viral loads greater than 1,000 copies/ml (>3 log_10_ copies/ml). The difference between the means was −1.06 log_10_ copies/ml (95% CI, −1.17 to −0.97; *P* < 0.0001) (Fig. 1b). The 95% limits of agreement (LOA) were −0.08 (95% CI, −0.25 to 0.09) log_10_ copies/ml and −2.16 (95% CI, −2.32 to −1.99) log_10_ copies/ml.

We evaluated the sensitivity of DBS to detect viremia at instrument detection thresholds as well as at WHO-defined thresholds for treatment failure of 1,000 copies/ml (30) and the previously defined threshold of 5,000 copies/ml (3). Using a threshold of instrument detection of viral particles, the sensitivity, specificity, positive predictive value (PPV), and negative predictive value (NPV) were 77%, 100%, 100%, and 78.5% (Table 1). At a viral threshold of >1,000 copies/ml for virologic failure the sensitivity, specificity, PPV, and NPV were 92.7%, 100%, 100%, and 94.3%, respectively (Table 1). Using a treatment failure threshold of VL >5,000 copies/ml led to a decrease in the sensitivity of the assay. The sensitivity, specificity, PPV, and NPV were 70.9%, 100%, 100%, and 82%, respectively (Table 1).

**TABLE 1:** Sensitivities, specificities, positive predictive values, and negative predictive values for treatment failure at different viral load thresholds

Threshold	Sensitivity (% [95% CI])	Specificity (% [95% CI])	PPV (% [95% CI])	NPV (% [95% CI])	Downward misclassification (%)	Upward misclassification (%)
TND/BLQ^*a*^	77 (69.4–85.5)	100 (97.1–100)	100 (96.8–100)	78.5 (71.2–84.6)	23	0
1,000 copies/ml	92.7 (88.6–96.6)	100 (97.6–100)	100 (96.8–100)	94.3 (89.5–97.4)	7.30	0
5,000 copies/ml^*b*^	70.9 (61.8–79)	100 (97.6–100)	100 (95.7–100)	82 (75.8–87.2)	29.10	0

a TND, target not detected; BLQ, below the limit of quantification.

b Sensitivities and specificities were calculated using both a plasma and DBS threshold of 5,000 copies/ml for the definition of treatment failure.

## DISCUSSION

Extraction of viral nucleic acids from dried blood spots results in an analyte that contains both cell-free and cell-associated viral DNA and RNA, providing erroneously high viral load values, particularly in samples with plasma values lower than 1,000 copies/ml (18–21). This has led to poor specificity for the detection of treatment failure in DBS specimens using the Amplicor or CAP/CTM systems (18, 20, 21). The resulting high false-positive rate at lower virus loads limits the use of this assay for routine viral load monitoring by DBS. PBS extraction of DBS samples results in the selective elution of cell-free virus (24), preventing overquantification of viral nucleic acids at low viral copy numbers compared with those of plasma. We used PBS elution from DBS, and show that this elution method significantly improved specificity at all thresholds evaluated.

The benefits of DBS testing may be tempered by discordances with plasma results across the higher RNA thresholds tested. At a threshold of 1,000 copies/ml, of the 9 discordant specimens, 2 were TND and 7 were BLQ by DBS, although higher than 1,000 copies/ml by plasma (reaching as high as a plasma VL of 34,160 copies/ml). This underclassification of treatment failure may represent a potential technical failure of the dried blood spotting and/or extraction processes. Continuation of a failing regimen is associated with poor long-term clinical outcomes, including increased mortality, accumulation of resistance mutations, and decreased likelihood of suppression on second line therapy (31–34). Although PBS can serve as a lower cost, simpler analyte for the extraction of viral load from DBS using the cell-free virus elution method (24), a normalization factor to include intrinsic differences between DBS and plasma and improvement in the extraction and amplification of viral RNA from DBS are warranted. A limitation of our study is that it was done in a controlled environment with dried blood spotting performed in the laboratory. Field testing with nurses or allied health care workers creating the DBS from finger pricks could result in changes in test sensitivity.

DBS viral load values were 1 log_10_ copies/ml lower than those obtained by plasma despite, including a software integrated correction for sample volume (22) and additional correction for the plasma fraction of whole blood. The bias that we noted is consistent with other studies showing that DBS viral loads are generally lower than plasma values (19, 35). We noted a higher mean difference than has been described in previous studies where the mean difference between DBS and plasma VL has ranged from −0.77 to +0.65 log_10_ copies/ml (14). This may be due to more selective elution of cell-free viral RNA only. A previous study of DBS for EID included a hematocrit correction factor that reduced the difference between DBS and plasma VL from −0.43 to −0.127 log_10_ copies/ml (36). We did not correct for serum hemoglobin levels but anticipate that the impact would be minimal, as a correction for the plasma fraction of whole blood was included. The −1 log_10_ copies/ml difference between DBS and plasma testing seen here is consistent with recommendations to provide a normalized DBS virus load for routine clinical practice. In resource-limited settings, all VL results greater than 1,000 copies/ml from either plasma or DBS should trigger intensification of adherence interventions with repeat testing at intervals to determine the appropriate management strategy.

Recent cost-effectiveness modeling analysis suggests that viral load testing using DBS and a reduction in scheduled visits among those who are virologically suppressed could be a more cost-effective strategy for monitoring patients on ART (37, 38). However, the high false-positive rate with DBS-based testing (21) that had been previously reported limited its use for routine viral load monitoring. Our data show that elution with PBS reduces the false-positive rate for DBS compared with that for plasma at a VL threshold lower than 1,000 copies/ml. These data have implications for national programs that use the Roche CAP/CTM platform for EID and plasma viral load monitoring, as this suggests that these platforms can be used to scale up viral load monitoring by DBS. However, improving the sensitivity of the assay by alterations in the buffer or changes in the methodology to use more than one spot should be considered and investigated further.

There was very good agreement, sensitivity, and specificity between DBS and plasma evaluated at the 2013 WHO-recommended plasma threshold of 1,000 copies/ml. DBS can be used for viral load monitoring; however, we would recommend a threshold for virologic failure of >1,000 copies/ml and not the previously recommended DBS threshold of 3,000 to 5,000 copies/ml (3). Collection of DBS for viral load monitoring on the CAP/CTM platform is already in use for EID of HIV and could enable national treatment programs to rapidly scale up access to HIV viral load testing in remote sites. Additional field-based testing would be necessary to ensure that the same high sensitivities and specificities at a threshold of 1,000 copies/ml can be achieved.

## MATERIALS AND METHODS

### Sample processing.

Consecutive specimens from pediatric and adult patients enrolled in research protocols at the University of Zimbabwe Department of Medicine that involved viral load monitoring were analyzed. Viral load assays were conducted at the University of Zimbabwe Department of Medicine Infectious Disease Research Laboratory. The laboratory has an internal quality assurance protocol for viral load testing, and participates in the Centers for Disease Control (Atlanta, GA) External Quality Assurance program for HIV viral load quantification. Fresh blood was collected in EDTA tubes. Dried blood spots (DBS) were prepared from whole blood prior to plasma separation. Fifty microliters (50 μl) of whole blood were spotted into three circles on DBS Whatman 903 card using calibrated pipettes and air-dried in laminar flow overnight. Cards were stored in individual zip-lock bags at room temperature with desiccant for up to 1 month prior to testing. EDTA plasma was obtained following centrifugation and stored at −80°C until processing.

Viral load was measured in plasma and DBS using the automated CAP/CTM assay according to the manufacturers' instructions. For plasma samples, 1.1 ml of plasma was transferred into the Cobas Ampliprep specimen preparation tube and loaded onto the analyzer using the HICAP 96 protocol (Roche Diagnostics Ltd., Rotkreuz, Switzerland). For the DBS one full circle was punched and placed into the Cobas Ampliprep specimen preparation tube and 1 ml of magnesium- and calcium-free phosphate-buffered saline (PBS: 1.54 mM NaCl, 5.6 mM Na_2_HPO_4_, 1.1 mM KH_2_PO_4_, pH 7.4; Corning Cellgro) extraction buffer was added to the tube and incubated at room temperature for 1 h without shaking. Without removal of the DBS paper, the tube was loaded onto the analyzer and processed using the H12DFSP96 protocol that was preinstalled by the instrument manufacturer (Roche Diagnostics Ltd., Rotkreuz, Switzerland). The CAP/CTM software reported a lower limit of quantification of <400 copies/ml. A correction factor of 2.8 was applied to account for the volume of whole blood applied to the DBS (50 μl) and an estimated plasma fraction of at least 50% (half of the whole blood).

### Statistical methods.

Data were analyzed descriptively and viral load values were log_10_-transformed for analysis. The Pearson correlation coefficient was used to measure the correlation between values obtained by DBS and those obtained by plasma.

The paired *t* test was used to compare mean values between DBS and plasma processing of specimens. The concordance between DBS and plasma specimen results was assessed using Bland-Altman analysis to calculate and plot the bias or mean difference and 95% limits of agreement, using log_10_-transformed data. Specimen pairs in which the VL was lower than 1,000 copies/ml (< 3.0 log_10_) by either method were excluded from the Bland-Altman analysis, as these included results that are not quantified, such as “target not detected” (TND) and “below the limit of quantification” (BLQ).

Agreement of the results for detection of virologic failure using thresholds of TND/BLQ (reported as either BLQ or TND), 1,000 copies/ml, and 5,000 copies/ml were determined using kappa statistics. We applied the Landis-Koch interpretation scale (kappa values of <0.40 indicate poor agreement; >0.40 and <0.75, fair to good agreement and >0.75, excellent agreement).

Using the plasma viral load as a reference, the clinical sensitivities, specificities, and negative and positive predictive values with 95% confidence intervals (CIs) were calculated to assess the performance of CAP/CTM DBS for detecting diagnostic thresholds TND, BLQ, <1,000 copies/ml, and <5,000 copies/ml. Clinical decision-making often does not distinguish between TND and BLQ; therefore, these two categories were analyzed as a single group (TND/BLQ). Standard definitions of sensitivity and specificity were used. The proportion of individuals with virologic failure at each threshold by plasma who were also detected as having virologic failure by DBS was used to determine the sensitivity. The proportion of individuals without virologic failure by plasma at each threshold who were correctly defined as not having virologic failure by DBS was used to determine the sensitivity. The downward misclassification documented the false-negative rate, and the upward misclassification the false-positive rate. All data were analyzed using STATA statistical software (version 13). Differences were considered significant when *P* values were less than 0.05.

### Ethics statement.

Written informed consent for viral load monitoring was obtained for all study participants. For children aged 12 or younger, informed consent was obtained from the legal guardian; for children aged 13 to 17, consent was obtained from the legal guardian and assent was also obtained from the child; adults and adolescents older than 18 years gave informed consent. The study protocols were reviewed and approved by the local institutional review board of the Joint Research and Ethics Committee of the University of Zimbabwe College of Health Sciences and Parirenyatwa Hospital, the Medical Research Council of Zimbabwe, and Partners HealthCare Human Research Committee.
